# Laser Doppler Imaging for Treating Vascular Complications from Procedures Involving Dermal Fillers: Case Series and Literature Review

**DOI:** 10.3390/diagnostics11091640

**Published:** 2021-09-07

**Authors:** An-Li Lee, Yu-Fan Chen, Wen-Teng Yao, Ying-Chun Liu, Chia-Meng Yu, Chieh-Ming Yu, Chih-Peng Tu, Wen-Chen Huang, Kwang-Yi Tung, Ming-Feng Tsai

**Affiliations:** 1 Division of Plastic Surgery, Department of Surgery, Mackay Memorial Hospital, Taipei 10449, Taiwan; ilive4308@gmail.com (A.-L.L.); vanadium29@hotmail.com (Y.-F.C.); wentyao@hotmail.com (W.-T.Y.); roctordoctor@gmail.com (Y.-C.L.); defense@mmh.org.tw (C.-M.Y.); jimmy_cmc36@yahoo.com.tw (C.-M.Y.); zaizen.medic@gmail.com (C.-P.T.); huang42@ms2.mmh.org.tw (W.-C.H.); tung@ms1.mmh.org.tw (K.-Y.T.); 2 Medical Cosmetic Center, Mackay Memorial Hospital, Taipei 10449, Taiwan; 3 Burn Center, Mackay Memorial Hospital, Taipei 10449, Taiwan; 4 Department of Surgery, Mackay Memorial Hospital, Taipei 10449, Taiwan

**Keywords:** dermal filler complications, intravascular injection of dermal filler, laser Doppler imaging, LDI

## Abstract

Vascular occlusion is a rare but severe complication of dermal filler injections. Early treatment of this complication produces better outcomes. Current diagnostic methods for vascular occlusion in the skin are subjective and imprecise; these include capillary refill time, skin color, and reports of pain. This study aimed to assess the use of laser Doppler imaging (LDI) in the evaluation and treatment of vascular complications caused by dermal filler injections. This retrospective study used laser Doppler imaging (LDI) in 13 patients who developed vascular occlusion after facial dermal filler injections, with subsequent follow-up. The precise areas of perfusion observed on LDI were compared with the findings of clinical and photographic evaluation. The results showed that LDI accurately identified areas of vascular occlusion and improved treatment precision among these thirteen patients. The procedure was more precise than visual inspection or photographic evidence. Satisfactory outcomes were achieved for all patients, and no procedure-related complications were reported. Collectively, LDI provides fast, noninvasive, and accurate delineation of areas of vascular occlusion caused by complications of dermal filler injections and avoids several subjective shortcomings of visual and photographic evaluations. Thus, LDI effectively tracks treatment outcomes. However, large-scale studies are required to confirm the present findings.

## 1. Introduction

Dermal filler injections are used widely in cosmetic surgery to fill deep lines and wrinkles and augment tissue [1], and their use has increased dramatically in recent years. In 2017, the American Academy of Plastic Surgeons reported 2.7 million procedures involving dermal filler injections. This represented a 3% increase over the previous year and an increase of over 250% from 2000 [2]. As many as 71% of these procedures involved the use of hyaluronic acid (HA)-based fillers in 2013 [3]. However, the increased use of dermal fillers has been associated with a growing frequency of complications related to their use [4]. Fortunately, cases of vascular complications arising from procedures involving dermal filler are extremely rare. Chiang et al. [3] reported anecdotally that out of 18,000 such procedures, approximately 10 patients (0.056%) developed vascular complications. Other authors have also suggested a vascular complication rate of 0.09% for procedures involving collagen implants [5]. The rate of inadvertent intravascular injection has been estimated as 3 per 1000 with calcium hydroxylapatite and 3–9 per 10,000 with HA-based products [6]. The true incidence of vascular complications may be higher, however, because of clinician underreporting [7,8]. The rarity of such complications makes it difficult to reach consensus on the diagnosis and treatment of vascular complications of procedures involving dermal fillers.

Arterial occlusion is one of the most common vascular complications caused by dermal filler injections and often occurs in regions perfused by a single artery, including the glabella, retina, alae nasi, and upper lip [8]. In the facial area, the arteria angularis (and its branches) is one of the most commonly affected arteries [6]. If the main blood vessel supplying an area of skin is obstructed by a filler agent, the associated vascular territory will develop ischemia and necrosis [6]. Arterial occlusion resulting from inadvertent intravascular injections is characterized by several symptoms, the most prominent of which is immediate pain accompanied by blanching and livedo reticularis (mottling). In cases when the retinal artery is affected, patients may exhibit monocular blindness [9,10]. Until recently, visual examination or photographic evidence was the common method for identifying the areas of arterial occlusion; however, precision was lacking for delineating the hypoperfused region. Consequently, a real-time, quick, low-cost, and accurate method is urgently needed to identify the suspected arterial occlusion after dermal filler injections.

Intravascular ultrasound has been used as a complementary imaging modality to angiography to provide structural information of the coronary arteries with high spatial resolution. This information includes lumen and vessel dimensions, plaque morphology, and location, thus enabling determination of the degree of stenosis [11]. Additional studies reported that high frequency ultrasound imaging was a potential method for identifying the suspected tissue damage and vascular complications in patients at risk of pressure ulceration [12,13,14]. Porter-Armstrong et al. explored whether ultrasound images supported clinical skin assessment in an inpatient population by identifying subcutaneous tissue damage [13]. However, the study demonstrated that qualitative classification of ultrasound images did not match outcomes yielded through clinical skin assessment [13]. In addition, no current consensus exists on tools by which to differentiate true ischemic zones from regions with inflammatory reactions or post-procedure subcutaneous hematoma. Moreover, no reliable methods exist for evaluating the effectiveness of salvage management. Laser Doppler imaging (LDI) is employed to measure superficial skin perfusion. LDI is a modality based on tracking diffusely reflected light over time; it can be used for perfusion imaging of the skin [15] and provides the advantages of portability, low-cost, and real-time imaging [16,17]. Additionally, LDI has been used for evaluating skin inflammation [18], wound healing, and rheumatic-associated vascular disorders [19], as well as in vivo pharmacological studies of human skin microcirculation [20] and the assessment of burn depth [21,22,23].

In LDI, a movable mirror is used to direct a laser beam onto the skin, so that direct contact is unnecessary. The movement of red blood cells is then rendered as a two-dimensional image [20] in which the area with reduced flow indicates the most hypoperfused area and the target area for treatment. The physician only needs to perform needling or to inject hyaluronidase into the embolic angiogram to become precisely detected by the device. Accurate delineation of the hypoperfused region limits the area subjected to needle punctures and hyaluronidase dose, resulting in less discomfort for the patient and better outcomes.

To the best of our knowledge, the present study is the first to assess the use of LDI in the evaluation and treatment of vascular complications caused by dermal filler injections. We hypothesized that LDI could reliably identify skin perfusion defects without interference from other factors. Toward this end, we compiled a cohort of thirteen patients with vascular complications for whom LDI was used to identify skin perfusion defects and guide treatment.

## 2. Materials and Methods

### 2.1. Study Setting and Design

This case series of patients treated over a 30-month period included consecutive patients who had received inadvertent intravascular injection with filler material, subsequently developed skin ischemia, and were transferred to Taipei Mackay Memorial Hospital between May 2018 and October 2020. The hospital’s institutional review board (IRB registration at 5 January 2018: 18MMHIS137e) approved the study protocol, and all patients provided signed informed consent.

Patients who developed cyanotic skin changes after facial dermal filler injections were included in this study, whereas those without significant evidence of inadvertent intravascular injection after facial dermal filler injection or of skin perfusion defects on the first LDI measurement were excluded. A total of thirteen patients were included, all of whom had developed early-stage vascular complications following treatment in cosmetic clinics.

### 2.2. Pre-Intervention Treatment Evaluation

The following details were recorded from the patients’ history at admission: medical history, location and type of dermal filler injection, and number of days between dermal filler injection and admission. All patients underwent clinical examination, facial photography, and LDI.

### 2.3. LDI Procedure

All patients underwent initial LDI using a Moor LDI device (MoorLDI2-IR; Moor Instruments, Axminster, UK) on the first day of admission. The device was placed at a distance of 57 cm from the patient, and images were obtained at a temporal resolution of 4 ms/pixel. Since inpatient LDI was only available during the daytime on weekdays, patients who had been hospitalized at night or on weekends underwent the procedure on the next working day.

### 2.4. Needling Procedure

Revascularization therapy was performed immediately after the completion of imaging through needling, in which 25-G needles (BD PrecisionGlide^TM^, Franklin Lakes, NJ, USA) were used to puncture blood vessels in the area of vascular occlusion. The aim was to ensure the extravasation of dermal filler within blood vessels by puncturing those that had been affected. The area with the most reduced perfusion on the LDI image was punctured several times or until bleeding was noticed. If the puncture hole began to rebleed, indicating a therapeutic effect, puncturing was stopped in that area and continued in other suspicious areas. This procedure was performed with or without hyaluronidase injection. If no substantial bleeding occurred, or if little injected material was seen in the pinhole of the hyaluronidase needle, the area was punctured more times and hyaluronidase injection was continued.

### 2.5. Ancillary Therapies

Continuous intravenous prostaglandin E1 (Ono Pharmaceutical Company, Osaka, Japan) infusion, intravenous and/or oral steroids, nonsteroidal anti-inflammatory drugs, and prophylactic oral antibiotics (amoxicillin/clavulanate) were among the ancillary treatments provided [24]. After completion of the treatment, cilostazol was administered to the patients for 7 days.

### 2.6. Post-Treatment Evaluations

Following LDI, local treatment (hyaluronidase and needling) was administered according to the skin perfusion defect visualized by the images. After the completion of local treatment, ancillary therapy was resumed. Subsequent follow-up imaging was performed every 1–3 days, depending on the clinical findings. If the previously punctured area continued to exhibit reduced flow, needling was performed again, with or without hyaluronidase. Subsequent follow-up imaging was not performed if LDI showed revascularization of the areas with reduced flow or if there was clinical confirmation of irreversible necrosis of the skin. All patients were photographed before each imaging round.

Follow-up LDI was continued until perfusion was completely restored or until the skin was confirmed to be necrotic. Photographic evaluations were continued until the facial skin had completely returned to normal.

## 3. Results

### 3.1. Patients’ Characteristics

Patients’ demographics and clinical features are described in Table 1. The study cohort included twelve females and one male who had experienced skin ischemia after undergoing facial filler injection (calcium hydroxylapatite: 1, HA: 12). The mean patient age was 38 years (range: 23–61, median: 40). In all, two patients were admitted to the study hospital for treatment only hours after receiving filler injection (Day 0), three patients were admitted on Day 1, three on Day 2, one on Day 3, two on Day 4, and two on Day 6 after the filler injection. The ischemic areas were nasal tip + bilateral nasal ala + glabella (*n* = 1), nasal tip + bilateral nasal ala (*n* = 1), nasal tip + ipsilateral nasal ala + ipsilateral mouth angle + ipsilateral nasolabial fold (NLF) + glabella (*n* = 1), ipsilateral nasal ala + ipsilateral upper lip (*n* = 1), upper and lower lips (*n* = 1), ipsilateral face necrosis (*n* = 1), nasal tip + bilateral nasal ala + ipsilateral NLF + upper lip (*n* = 1), ipsilateral nasal ala + ipsilateral NLF (*n* = 1), nasal tip + bilateral nasal ala +ipsilateral NLF (*n* = 1), ipsilateral nasal ala + ipsilateral NLF (*n* = 1), nasal tip + ipsilateral nasal ala + ipsilateral NLF + ipsilateral nasal dorsum (*n* = 1), ipsilateral forehead + ipsilateral temporal fossa + ipsilateral preauricular region + ipsilateral upper eyelid ptosis (*n* = 1), and nasal tip + columella (*n* = 1) (Table 2). Patients 4, 5, 6, and 7 had received repeated hyaluronidase therapies before they were admitted to the study hospital. All patients exhibited partial facial skin cyanosis, which had probably been caused by the inadvertent intravascular injection of dermal fillers. The cyanosis involved the nose, glabella, and lips in most patients. In addition to the cyanotic areas seen on the photographs, most of the areas involved were accompanied by pain and/or numbness. LDI was performed as soon as possible after admission. Following examination, local treatment of the perfusion defect area was performed with needling or hyaluronidase injection, according to the LDI findings. As ancillary therapy, PGE1, steroids, antibiotics, nonsteroidal anti-inflammatory drugs, or antibiotic creams were administered for wound care, as described in Table 2.

### 3.2. LDI Provides an Accurate Delineation of the Hypoperfused Region

**Figure 1:**
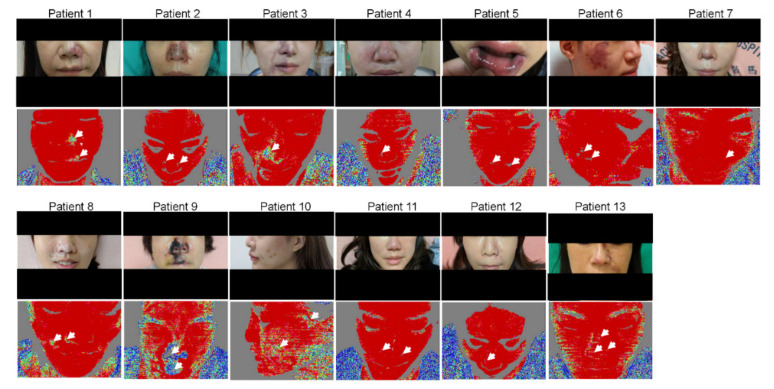
Clinical photographs and laser Doppler imaging of all patients. At Day 1 of admission, the clinical photographs and DPI represent arterial occlusions in the region of facial filler injection (closed arrows).

**Figure 2:**
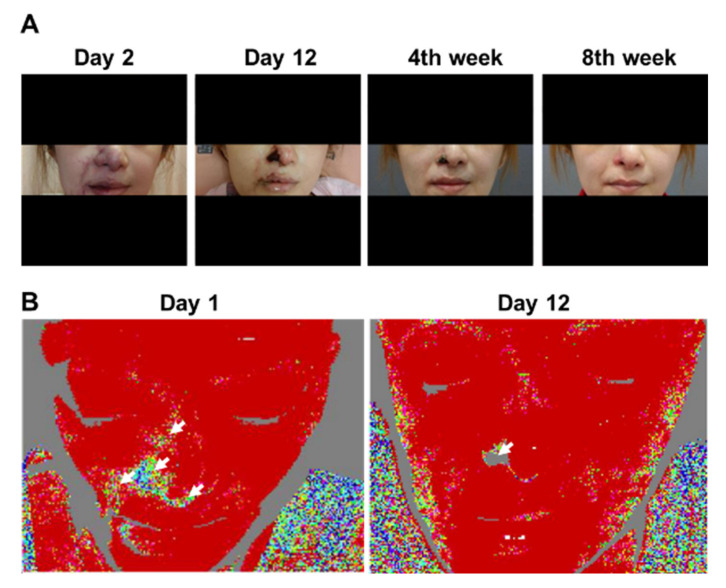
Clinical photographs and laser Doppler imaging of Patient 3. (**A**) Day 2 of admission; Day 12 of admission; Week 4 of follow-up; Week 8 of follow-up. Initial worsening and gradual improvement of cutaneous symptoms can be observed. Finally, only the right ala exhibited thick skin necrosis, which left an obvious scar. (**B**) Left panel: on Day 1 of admission, areas of cutaneous ischemia were clearly delineated from those without vascular occlusion. Right panel: on Day 12 of admission, although the originally affected area had not returned to its normal state of appearance, blood circulation of the other parts, except for the right ala, was restored.

**Figure 3:**
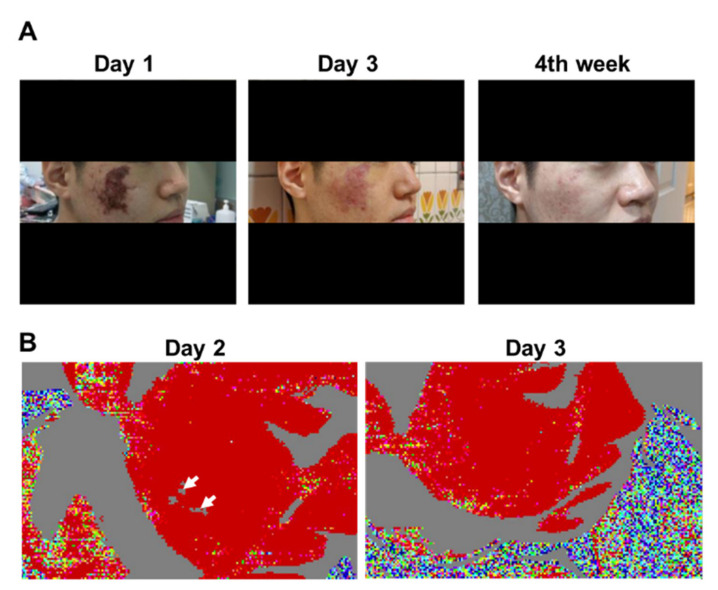
Clinical photographs and laser Doppler imaging of Patient 6. (**A**) Day 1 of admission; Day 3 of admission; Week 4 of follow-up. Gradual improvement in cutaneous symptoms can be observed. (**B**) Left panel: on Day 2 of admission, areas of cutaneous ischemia were clearly delineated from those without vascular occlusion. Right panel: on Day 3 of admission, facial blood circulation was restored.

On Day 1 of admission, the region of the vascular occlusions in patients was assessed by LDI. As shown in Figure 1, LDI accurately showed the hypoperfused region in all patients. The needle punctures and hyaluronidase doses were performed based on the LDI findings in each case. After the treatment, the results of LDI exhibited improvement of the vascular occlusion at the hypoperfused region in these patients (Table 2 and Figure 2 and Figure 3). Moreover, we did not encounter complications from the procedure in any of the patients after 30-month follow-up. Furthermore, detailed results of treatment for two specific cases are described below.

### 3.3. Patient 3

A 31-year-old female with a history of open rhinoplasty and septal reconstruction with prosthesis 1 year previously was admitted with a wide range of cyanotic skin changes immediately after undergoing bilateral NLF HA injections (1 mL bilaterally) via a 25-G cannula.

The treating clinician immediately performed needling and hyaluronidase injection at the site and transferred the patient to our emergency department. Physical examination revealed cyanotic changes of the nasal tip, right nasal ala, right NLF, right mouth angle/right upper and lower lips, and right glabella. Because the patient was admitted late at night, ancillary therapy with PGE1, steroids, and antibiotics was administered. The following day, LDI was performed, revealing perfusion defects in the right nasal ala, right NLF, and right lower lip. Needling and hyaluronidase injection were performed. Over the next 3 days, the cyanosis in her right NLF and right upper and lower lip was resolved; however, the right ala cyanosis gradually became gangrenous. Although PGE1 was continued, her symptoms did not improve. Several LDI procedures were performed in succession, and perfusion defect was noted only in the left right ala. She was observed for 15 days after admission, before the right ala skin was judged to have undergone full-thickness necrosis. After the other areas were treated, the patient was discharged with a 1-week prescription of cilostazol and antibiotics. The rest of the areas recovered well after discharge; however, the right ala skin healed after approximately 2 months, producing a more pronounced scar contracture (Figure 2).

### 3.4. Patient 6

A 25-year-old male with no remarkable previous medical history was admitted exhibiting gangrenous changes on his right cheek 6 days after receiving HA injection (1 mL, 27-G needle) for the treatment of acne scars. He reported noticing skin changes after the filler injection without realizing the problem until the sixth day after treatment. The treating clinician had performed needling and hyaluronidase injection into the entire gangrenous area before the patient was urgently transferred to our emergency department. His lesion was judged to be a complication of inadvertent intravascular injection of the filler, and LDI was performed the following day. Although his appearance and photographs revealed a large gangrenous area, LDI merely revealed several small perfusion defects on the right cheek. Needling was performed only in the areas where LDI showed perfusion defects. PGE1, steroids, and antibiotics were administered as ancillary therapy. LDI was repeated on the third day after admission, and the perfusion defects were confirmed to have resolved completely. However, remnants of sporadic skin erosion were evident on visual and photographic examinations of the right cheek. The patient was discharged with a 1-week prescription of cilostazol and antibiotics. During follow-up visits 4 weeks after discharge, the wound was found to be healing, leaving only a small scar and PIH (Figure 3).

## 4. Discussion

All thirteen patients in the present case study were managed initially by the treating clinician, primarily with or without hyaluronidase injections in addition to other treatments prior to admission to the Taipei Mackay Memorial Hospital. Using LDI and needling, in all patients were accurately located the ischemic areas affected by dermal filler emboli and achieved satisfactory outcomes. Although two patients demonstrated skin necrosis, we did not encounter complications from the procedure in any of the other cases. Results of these cases suggest that LDI can be used to determine precisely the target area for treatment and to trace its effects. LDI allowed the accurate identification of areas with vascular occlusion following inadvertent intravascular injection of the dermal filler material with greater precision than visual examination or photographic evidence. These findings thereby support our initial hypothesis that LDI can reliably identify skin perfusion defects without interference from other factors.

Inadvertent intravascular injection of dermal fillers causes emboli, leading to skin necrosis and unpleasant scarring if these emboli are not treated in time. The progression of clinical skin ischemia is typically used to judge the progress of the disease in such patients, although this method is subjective and inaccurate. The use of LDI to confirm perfusion defects overcomes most of the shortcomings of visual and photographic examinations.

During the study period, a young female patient who was not included in the study cohort felt discomfort at the left nasal dorsum after undergoing dermal filler injection for nasal augmentation. She also experienced a dull, mild pain at the site. Due to concern over inadvertent intravascular injection of the dermal filler, she visited our clinic for consultation. We performed LDI and ruled out perfusion defects, and we discharged her with instructions for follow-up. No problems were reported during follow-up over 7 days. The original dullness of the affected area of skin also improved (data not shown). Although this was our experience in one single case, we suspect that, besides identifying perfusion defects, the use of LDI may help clinicians avoid unnecessary procedures for patients without true vascular occlusion.

Complications of dermal filler injections can be classified as technical or inflammatory [25]. Technical complications include errors such as excessive or inadequate volume of injection, overly shallow or deep injection, and incorrect location or product. Inflammatory complications include infections and immunological reactions to the product. Of these, inadvertent intravascular injection can be considered a rare technical error, though it has potentially devastating consequences. The signs and symptoms of inadvertent intravascular injection and embolization of facial arteries include blanching or dusky coloration of the skin, ecchymosis, reticulated erythema, and intense pain in the treated area [26]. Among the included patients, pain and skin discoloration were common signs and symptoms. The areas at high risk of vascular occlusion and necrosis are the glabella, nasal ala, cheeks, perioral region, and temples. Of these, the glabella carries the highest risk because the vessels are the thinnest, and the area has the least collateral circulation [26]. In the patient cohort of the present study, the glabella, nasal ala, and lips were most often affected. Intra-arterial injection of dermal filler can occur when using either needles or cannulas. With the latter, the inadvertent intravascular injection is often because it is impossible for the practitioner to perceive the difference between penetrating a fibrous septum and an artery when performing a blind puncture [27]. Consequently, LDI may be the most likely method to identify the occurrence of skin effect of dermal filler-caused inadvertent intravascular injection. Certainly, it merits further investigation.

The risks associated with intravascular injection of dermal fillers can be reduced by undertaking the following precautions [28]: (1) aspirating from the needle or cannula prior to the injection to verify that there is no backflow of blood; (2) using small volumes and administering several small injections in high-risk areas such as the glabella; (3) injecting in a more superficial plane; (4) treating only one side at a time; (5) pinching and/or tenting the skin to create increased space superficial to the branches of the main arteries, and (6) compressing the origin of the supratrochlear vessels with a nondominant finger. In specifically sensitive regions such as the glabella, it is recommended that very low volumes of filler be used [29]. Failure to do so can result in intravascular retention of the filler, generating necrosis and a foreign-body inflammatory response that is visible on biopsy. Appropriate patient selection is an accepted method of reducing complication rates of various medical and surgical interventions. De Boulle et al. [30] provided a list of patient conditions that might serve as absolute or relative contraindications to dermal filler injections. Nevertheless, it is not clear whether any preexisting condition would predispose a patient to vascular occlusion through the injection of dermal fillers. Indeed, none of the patients in the present series possessed remarkable previous medical histories. In addition, in-depth knowledge of vascular anatomy is essential to reduce the risk of inadvertent intravascular injection of dermal fillers. A specific body of evidence for aesthetic medicine practitioners is gradually being accumulated that can help inform them and avoid vascular complications. For example, Cotofana et al. [31] performed a comprehensive cadaveric study of the superficial vasculature of the lip that was designed to provide clinicians with a framework for avoiding vascular occlusion. Additionally, a practitioner’s years of experience appear to correlate with the lifetime risk of performing an inadvertent intravascular injection [32]. Among practitioners with more than 11 years’ experience, the majority (62%) reported having performed one or more inadvertent intravascular injections [32]. In addition, less experience did not appear to correlate with greater risk of this complication.

Absolute risk reduction may be theoretically impossible. In fact, Wang et al. [33] suggested that because of the considerable degree of vascular variability, a “zero-risk” zone does not prevail. This aspect should provide more impetus to prepare for the occurrence of vascular occlusion and appropriate early therapy.

Skin perfusion can be evaluated using several modalities, including clinical examination for skin color change, capillary refill time, or relief of pain, as well as photography and other imaging modalities [34]. However, clinical examination and photography at pre-and post-treatment are subjective and an imprecise means of evaluating skin perfusion [34]. Other imaging modalities include thermography, video-microscopy, and spatial frequency domain imaging [34]; however, clinical trials have not demonstrated the effectiveness of these methods [34]. Ultrasound is a relatively simple and noninvasive imaging modality that can be used to identify the nature and location of dermal filler injections [35]. However, these modalities cannot identify acute intravascular complications. The previous study indicates that intravascular ultrasound-based angiography may fail to evaluate the severity of arterial lumen narrowing when there is no truly “normal” blood vessel that can act as a reference site [36]. Therefore, ultrasound would not serve as a diagnostic modality of choice in such circumstances. According to the results of the present study, LDI may be a potential method for identifying arterial occlusion.

Hourly high-dose pulsed hyaluronidase injection in the approximate ischemic area (determined clinically by the observation of skin color and capillary refill time) provides excellent results [37]. Without imaging, which helps to narrow down the affected areas, multiple needle punctures would be required over all suspicious areas, thus involving a relatively large area of tissue, and cause discomfort and damage to the skin. Sun et al. [38] reported a consecutive series of 20 patients exhibiting impending skin necrosis of the face, with most involving the nose. All were treated with hyaluronidase injection and ancillary therapy comprising antibiotics, tanshinones, papaverine, topical magnesium sulfate, infrared irradiation, hyperbaric oxygen, and aspirin (unless contraindicated). Of the 20 patients, 13 achieved complete resolution, and 7 exhibited skin necrosis. The authors attributed the treatment failure to delayed treatment (>2 days after dermal filler injection). In addition, Han et al. [39] reported the case of a female with skin necrosis of the glabella 5 days after HA injection. Despite receiving appropriate therapy, scarring of the skin occurred. However, all patients in the present study were admitted within 48 h of the initiating event. Salval et al. [40] reported that a case of extensive midfacial necrosis in a patient after HA injection was treated successfully with intravenous corticosteroids and antibiotics [40]. Therefore, such evidence suggests that early intervention is essential to achieve favorable outcomes. Patients in the present series were managed with needling. Guided by LDI, this technique limited the areas of needle puncture to those directly affected by vascular occlusion, thereby avoiding unnecessary damage to normal tissue. The principle behind this therapy is to puncture the arteries embolized with dermal filler so as to release the material into the extravascular space, and thereby relieve obstructions. If the filler material is HA, concurrent hyaluronidase injections will help digest the material quickly and efficiently.

In cases with suspected vascular occlusion in the immediate post-injection period, several steps are recommended [41], including the immediate application of warm compresses to promote vasodilation, thus increasing local perfusion [42,43,44]. In some cases, particularly those caused by arterial compression rather than by intravascular emboli, warm compresses and massage may be adequate. Other studies have reported several alternative techniques for treating tissue ischemia. Kleydman et al. [45] recommend the use of nitroglycerin paste to improve local blood flow. Other treatment modalities include the use of vasodilators and hyperbaric oxygen [41]. According to DeLorenzi [37], however, the available evidence is not sufficient to recommend further use of hyperbaric oxygen, nitroglycerin paste, PGE1, or aspirin (although there remains at least a theoretical basis for continuing aspirin as a platelet aggregation inhibitor). Among these ancillary treatments, PGE1 was used in the present study cohort since it produces effects of both vasodilation and platelet aggregation inhibition. We believe that PGE1 can improve the ischemic changes caused by intravascular injection and help maintain skin perfusion during treatment. Nevertheless, we do not claim that a particular ancillary therapy contributed substantially to the recovery of the included patients. Indeed, the role of ancillary therapies should be evaluated in detail to settle the issue of whether they contribute to the management of post-injection vascular occlusion.

LDI is a rapid and easy-to-perform technique. Moreover, it is reliable and noninvasive in the context of treating vascular occlusion following inadvertent intravascular injection of dermal fillers. However, a disadvantage of LDI is that it may cause some dead angles of inspection if there is a significant height difference in the measured areas. In such situations, the problem can be solved by changing the angle of imaging. In previous studies on salvage procedures, we found several reported techniques used to treat or improve skin ischemia caused by intravascular injection of dermal fillers. However, only limited data are available on the appropriate treatment range, treatment end point, and evaluation techniques. This is the result of a lack of accurate, reliable, and instant assessment tools. Hence, LDI was employed in the present study to overcome these challenges.

### Limitations

This study has several limitations. First, it was a retrospective case series of patients who had been referred to our clinic after being treated as outpatients with dermal fillers. Therefore, the study findings are subject to selection bias. Nevertheless, the fact that our patients achieved favorable outcomes even after substantial time-lag following the initial event suggests that such LDI-guided needling performed in the treating clinician’s office would produce better effects than the results obtained in this series. This limitation may be overcome by a prospective study, preferably including clinicians involved at the initial point of care.

Second, some patients were reluctant to return for extended follow-up. Therefore, we cannot state with certainty that all included patients enjoyed favorable long-term outcomes. Nevertheless, given the nature of the problem being treated, the fact that these patients were all discharged with favorable outcomes strongly suggests that all perfusion defects were successfully resolved. A future study with long-term follow-up of at least 6 months would be necessary to validate the robustness of our findings.

Third, the sample size was small. This, combined with the fact that all patients showed satisfactory outcomes, made it impossible to perform statistical analyses of our results. Moreover, since intravascular injection of dermal fillers is an extremely rare event, the collection of enough cases from which proper statistical analysis can be performed may prove to be extremely difficult. Nevertheless, clinicians should be encouraged to record all such events and document outcomes for the benefit of their current and future patients.

Finally, the device we used (MoorLDI2-IR) was an old model. New models may provide faster examination efficiency and more detailed information. Nevertheless, given the positive outcomes in the present study, we believe that new models will generate even better results than those obtained in the present series.

## 5. Conclusions

LDI is fast, easy-to-perform, reliable, and noninvasive. It measures superficial microcirculatory perfusion in the skin and can precisely locate ischemic zones caused by dermal filler injection. It also allows the extent of local treatment to be determined after the inadvertent intravascular injection of dermal fillers, as well as evaluation of the therapeutic effect of salvage management with confidence. Nevertheless, these findings warrant validation in large-scale prospective studies with large cohorts.

## Figures and Tables

**Table 1:** Patient demographics and clinical features.

Patients	Age (Years)	Sex	Past History	Location and Details of Dermal Filler Injections	Type of Dermal Filler	Overall Follow-Up (Days)	Day of Dermal Filler Injection before Admission
Patient 1	40	F	Two NLF rejuvenations with PLLA in the past 3 years	NLF R/L 1 mL/1 mL (with 23-G cannula)	HA	14	D2
Patient 2	25	F	Nil	Nasal tip 0.3 mL/dorsum 0.3 mL/radix 0.3 mL/chin 0.6 mL (with 27-G needle)	CaHA	24	D4
Patient 3	31	F	Open rhinoplasty and septal reconstruction 1 year ago	NLF R/L 1 mL/1 mL (with 25-G cannula)	HA	53	D0 (6 h)
Patient 4	42	F	Nil	Nasal tip 0.2 mL/dorsum 0.1 mL (with 27-G needle)	HA	11	D4
Patient 5	45	F	Nil	Upper and lower lips 1 mL (with 27-G needle)	HA	13	D1
Patient 6	25	M	Nil	Acne scar treatment (multiple sites involving bilateral cheeks): Subcision followed-by HA injection (with 27-G needle): 1 mL totally	HA	20	D6
Patient 7	43	F	Close rhinoplasty with prosthesis	Nose augmentation 2 c.c. (with 27-G needle)	HA	13	D3
Patient 8	33	F	Nil	Bilateral NLF R/L: 1.6 c.c./0.4 c.c. (with 27-G needle)	HA	12	D0 (6 h)
Patient 9	61	F	Nil	Nasal tip 0.8 c.c. (with 27-G cannula)	HA	100	D6
Patient 10	23	F	Nil	Bilateral forehead and temporal fossa R/L: 3 c.c./3 c.c. (with 21-G cannula)	HA	10	D2
Patient 11	43	F	Nil	Bilateral NLF R/L: 0.4 c.c./0.4 c.c. Left tear trough: 0.3 c.c. (with 21-G cannula)	HA	28	D1
Patient 12	29	F	Nil	Bilateral NLF R/L: 0.4 c.c./0.4 c.c. (with 27-G needle)	HA	12	D1
Patient 13	47	F	Nil	Bilateral NLF R/L: HA 1 c.c./1 c.c. (with 22-G cannula)	HA	12	D2

CaHA, calcium hydroxylapatite; D, Day; F and M, female and male, respectively; HA, hyaluronic acid; NLF, nasolabial fold; OPD, outpatient department; PLLA, poly-L-lactic acid; R and L: right and left, respectively.

**Table 2:** Clinical examination, photography, and patients’ LDI findings on days 0, 3 and 14 of admission.

Patients	Day of or 1 Day after Admission		Three Days after Admission		Approximately 2 Weeks after Admission	
	Clinical/Photographic Findings	LDI Findings	Clinical/Photographic Findings	LDI Findings	Clinical/Photographic Findings	LDI Findings
Patient 1	Cyanotic change of the L nasal ala and the L upper lip	L nasal alar area partial perfusion injury	Few gangrenous changes of the L nasal ala	No perfusion defect	Few scars on and PIH of the L nasal ala	NA
Patient 2	Cyanotic change of bilateral nasal ala and nasal tip	R nasal alar perfusion defect	Gangrenous change of the R nasal ala and few cyanotic changes of L nasal ala and nasal tip	No perfusion defect	Few scars on and PIH of the R cheek	NA
Patient 3	Cyanotic change of the nasal tip, R nasal ala, R NLF, R mouth angle/R upper and lower lips, R glabellar region	R nasal ala, R NLF, R lower lip partial perfusion defect	Cyanotic change of the R nasal ala, R mouth angle/right upper and lower lips	R nasal alar perfusion defect	R alar eschar	No perfusion over R nasal ala
Patient 4	Ischemic change of the nasal tip/bilateral nasal ala/dorsum and R glabellar region	R and L nasal alar area perfusion defects	Ischemic change of the nasal tip/dorsum, gangrenous change of bilateral nasal ala	NA	NA	NA
Patient 5	Cyanotic changes and swelling of the upper and lower lips	NA	R oral ulcer (MBD)	No perfusion defect	Healing R oral ulcer	NA
Patient 6	R cheek skin necrosis	R cheek spot perfusion deformity	Healing skin necrosis of the R cheek	R cheek spot perfusion deformity disappeared compared with 2 days prior	Few scars on and PIH of the R cheek	NA
Patient 7	Cyanotic changes of the nasal tip and columella	NA	Few cyanotic changes and swelling of nasal tip	No perfusion defect	Minimal scar and PIH on nasal tip	NA
Patient 8	Cyanotic changes of the right nasal ala and right NLF	Right nasal ala and right NLF perfusion defect	Few cyanotic changes of the right nasal alar and right NLF	No perfusion defect	Few PIH on right nasal ala	NA
Patient 9	Ischemic changes of the bilateral nasal ala, nasal tip, right NLF, and upper lip	Bilateral nasal ala, nasal tip, right NLF, and upper lip perfusion defect	Gangrene changes of the bilateral nasal ala, nasal tip, right NLF, and upper lip	NA	Gangrene changes of the bilateral nasal ala, nasal tip, right NLF, and upper lip	NA
Patient 10	Cyanotic changes of left forehead, left temporal fossa, and left preauricular region. Left upper eyelid ptosis	Left forehead, left temporal fossa, and left preauricular region perfusion defect	Cyanotic changes of left forehead, left temporal fossa, and left preauricular region. Left upper eyelid ptosis	NA	Few PIH on left forehead, left temporal fossa, and left preauricular region.	NA
Patient 11	Cyanotic changes of the bilateral nasal ala, nasal tip, and left NLF	NA	Few cyanotic changes of the bilateral nasal ala and nasal tip	Bilateral nasal ala and nasal tip perfusion defect	Few PIH on nasal tip	NA
Patient 12	Cyanotic changes of the left nasal ala and left NLF	No perfusion defect	Few cyanotic changes of the left nasal ala and left NLF	NA	Few PIH on left nasal ala and left NLF	NA
Patient 13	Cyanotic changes of the nasal tip, left nasal ala, left nasal dorsum, and left NLF	Left nasal ala, nasal tip, and left nasal dorsum perfusion defect	Few cyanotic changes and swelling of the nasal tip, left nasal ala, left nasal dorsum, and left NLF	No perfusion defect	Few PIH on left nasal ala and nasal tip	NA

LDI: laser Doppler imaging; MBD: may be discharged; NA: not available; NLF: nasolabial fold; PIH: post-inflammatory hyperpigmentation; R and L: right and left, respectively.

## Data Availability

The data used to support the findings of this study are included within the article.
